# Hematologic alterations and early mortality in a cohort of HIV positive African patients

**DOI:** 10.1371/journal.pone.0242068

**Published:** 2020-11-10

**Authors:** Fausto Ciccacci, Francesca Lucaroni, Roberto Latagliata, Laura Morciano, Elisa Mondlane, Moises Balama, Dyna Tembo, Jane Gondwe, Stefano Orlando, Leonardo Palombi, Maria Cristina Marazzi

**Affiliations:** 1 UniCamillus, Saint Camillus International University of Health Sciences, Rome, Italy; 2 Department of Biomedicine and Prevention, University or Rome Tor Vergata, Rome, Italy; 3 Hematology, Department of Translational and Precision Medicine, University ‘Sapienza' and Policlinico Umberto 1, Rome, Italy; 4 DREAM program, Community of Sant’Egidio, Maputo, Mozambique; 5 DREAM program, Community of Sant’Egidio, Beira, Mozambique; 6 DREAM program, Community of Sant’Egidio, Blantyre, Malawi; 7 LUMSA, Rome, Italy; Katholieke Universiteit Leuven Rega Institute for Medical Research, BELGIUM

## Abstract

**Introduction:**

Infection with Human Immunodeficiency Virus (HIV) is highly prevalent worldwide, especially in Sub-Saharan Africa, where anaemia is also widespread. HIV infection is known to be associated with anaemia and various other haematologic alterations, but little data on correlation with immunological and virologic conditions in treatment-naïve patients and impact on mortality are available. Our study aims to investigate hematologic features in HIV-infected individuals in Malawi and Mozambique and assesses possible correlations with early morality.

**Material and methods:**

We conducted a retrospective analysis of baseline data (general details, nutritional status, full blood count and HIV infection progress data) and 12 months follow-up status for HIV+ adult patients in 22 health facilities in Malawi (11 sites) and Mozambique (11 sites) run by DREAM program. Anagraphic details, anthropometric characteristics, full blood count, CD4+ count and Viral Load data were collected from electronical medical records (EMR) for all the HIV-positive, treatment-naïve patients starting care in the sites in the period January 2007 –December 2016. Follow-up status after one year since enrolment in care was also considered. All the data extracted from the EMR were included in a dataset and then analysed. Univariate and multivariate analysis were conducted through logistical regression to investigate associations, and survival analysis analysed in a Cox regression model.

**Results:**

On the whole, 22.657 patients were included; severe and moderate anaemia were observed in 1.174 (8,2%) and 4.703 (21,9%) patients respectively. Gender, nutritional status, CD4+ count, and viral load (VL) were associated with anaemia, leukopenia, and thrombocytopenia. Among 21.166 fully evaluable patients, 8.494 (40,1%) had at least one cytopenia. Any cytopenia was present in 1/3 of patients with normal nutritional status and less advanced HIV infection, and it wouldn’t be diagnosed in a basic HIV care setting. During the first year of treatment, 1.725 subjects (7,6% of the entire sample) died. Anaemia, lower Red blood cells and platelets counts correlated with mortality in the first year of care, independently by body mass index, haemoglobin, CD4+ count and VL.

**Conclusions:**

Notwithstanding anaemia is known to be associated with HIV infection at diagnosis, full blood count is not routinely performed in many African countries. Our results emphasize that including the study of a broader set of parameters in the routine HIV care services in Sub-Saharan Africa would provide significant clinical information able to predict other alterations and poor outcomes.

## Introduction

Human Immunodeficiency Virus (HIV) infection is highly prevalent and represents one of the leading causes of morbidity and mortality in Sub-Saharan Africa [1]. Malawi and Mozambique are among the HIV most affected countries in the world: in Malawi, it is estimated that one million people are living with the virus [2], and that it has a prevalence of 9,6% in the adult population; in Mozambique the prevalence of HIV is 12,6% [3].

Anaemia is a widespread condition in the general population, especially in limited-resource countries; it is also associated with many comorbidities, reduced quality of life and reduced life expectancy [4]. The African region is particularly affected by anaemia [5], which can be caused by iron and/or nutritional deficiency, micronutrient deficiency (e.g. riboflavin, folate, A and B12 vitamins), acute and chronic infections (e.g. malaria, cancer, tuberculosis), and inherited or acquired alterations that affect haemoglobin synthesis, red blood cell (RBC) production or survival [6].

HIV infection is known to be associated with anaemia and various other haematologic alterations [7]; however, little data about correlation with immunological and virologic conditions in treatment-naïve patients are available [8].

Like many other national programs in resource-limited countries, Malawian and Mozambican national HIV-care programs do not usually offer hematologic tests to patients who are accessing the antiretroviral treatment (ART). As a result, no extensive real-life study is presently available in Africa to evaluate hematologic alterations that occur in patients living with HIV (PLWH) at a primary care level. Most of the studies published in the last decades involving HIV-positive African patients are focused on anaemia and haemoglobin evaluation, but scarce data are available about other haematological features [9–11].

The present study aims to highlight the incidence of hematologic alterations occurring in HIV-infected individuals in Malawi and Mozambique at the baseline of ART and identify possible impacts on early mortality.

## Materials and methods

### Patient population

We conducted a retrospective analysis of baseline routine data in PLWH consecutively enrolled between 1/2007 and 12/2016 by 22 DREAM (Diseases Relief through Excellence and Advanced Means) health facilities in Malawi [11] and Mozambique [11].

DREAM is a public health program run by the Community of Sant'Egidio in 11 SSA (sub-Saharan African) countries that focuses on HIV prevention, care and retention strengthening, TB, and other diseases [12–15]. Each patient accessing the centres managed by the DREAM program has access to routine haematologic tests (in addition to molecular and biochemistry analyses) from the baseline of ART through follow-up according to national and/or international guidelines. All the services and laboratory tests in the DREAM program are managed by a DREAM software that allowed to collect all the data needed for the analysis [16].

Data recorded before the start of ART and at 12 months after the start of treatment for all adult HIV+ naïve patients were collected and anonymized to remove recognizing details: each patient was identified by an alphanumeric code (ID), and anonymous data were included in the datasheet. The researchers received from the centres all the data already anonymized so that it could be not possible to identify patients.

Ethical approval of the protocol was achieved from the District Health Officer in Blantyre, Malawi (ref no: BT DHO/MED/9). As only routine data were analysed, informed consent was not required.

For each patient we included the following data: anagraphic details (age, gender, country), anthropometric characteristics [Body Mass Index (BMI)], full blood count, CD4+ count and Viral Load (VL).

### Definitions

Anaemia was defined as mild [haemoglobin (Hb) < 12 ≥ 10 g/dl], moderate (Hb < 10 ≥ 8 g/dl) or severe (Hb < 8 g/dL) [6]. In addition, anaemia was classified as one of three types according to the size of the RBCs: microcytic if associated with a mean corpuscular volume (MCV) of less than 80 fL, normocytic if RBCs were sized 80–100 fL and macrocytic if the MCV was higher than 100 fL [17]. Leukopenia was defined as a white blood cell (WBC) count < 4 x 10^9^/l, and thrombocytopenia was defined as a platelet (PLT) count < 100 x 10^9^/l.

Body mass index (BMI) was calculated as weight/(height)^2^, as indicated by WHO, and malnutrition was defined as BMI < 18,5kg/m^2^ [18].

HIV-infection stage at baseline of ART was evaluated according to clinical WHO classification [19].

### Statistical methods

#### Baseline analysis

Data were expressed as mean ± standard deviation (SD) (normally distributed data), median and interquartile range (IQR; non-normally distributed data), or as percentage frequencies, and within-patient comparisons were made by unpaired t-test and X^2^ test, as appropriate, at significance levels of p<0.05. Logistic regression models were used to compare baseline variables before follow-up in bivariate and multivariate analyses. Odds ratios with 95% confidence intervals (CI) were used to assess the strength of associations.

#### Survival analysis

A multivariate survival analysis evaluating premature death as an outcome was performed using a Cox regression model. Early mortality was defined as a fatal event occurring within the first 12 months of follow-up, whether receiving antiretroviral therapy or not. Cox analysis was adjusted for three parameters: VL (as a proxy of virologic progression), CD4+ cell count (as an immunologic marker of disease) and BMI (as a disease progression marker). Time was calculated from enrolment to 12-month follow-up or premature end of assistance [for death or lost to follow-up (LTFU)].

All analyses were performed using SPSS software, version 22 (IBM Corp, 2013, Armonk, NY, USA).

All the analysis will be conducted including only the patients with available data needed for the analysis.

## Results

### Baseline analysis

In total, 22.657 HIV+ treatment-naïve patients met the inclusion criteria for this study. The basic characteristics and haematologic features of the studied cohort are shown in Tables 1 and 2, respectively. The complete data were not available for all the cohort, hence the analysis were conducted involving the available data.

**Table 1:** Basic characteristics of the entire cohort.

Gender, n° (%): • Male	- 8.570 (37,8)
• Female	- 14.087 (62,2)
Median age, years (Inter Quartile Range)	35 (29–42,5)
WHO HIV Clinical stage, n° (%)*:• I	- 8.427 (48,8%)
• II	- 4.450 (25,5%)
• III	- 3.946 (22,6)
• IV	- 604 (3,6%)
Median Body Mass Index (kg/m2, median, IQR)**	20,9 (18,8–23,5)
Median CD4+ cell count, cells/mm3 (IQR)***	233 (122–334)
Median Plasma HIV-RNA, log10 copies/ml (IQR)****	4,44 (3,75–5,00)

* n = 17.427

** n = 19.008

*** n = 22.657

**** n = 17.001

**Table 2:** Haematological features of the cohort.

Median Red Blood Cells x 109/L (Inter Quartile Range)*	4,18 (3,70–4,66)
Median Haemoglobin, g/dL (IQR)**	11,2 (9,7–12,6)
Anaemia, n° (%): • Severe	1.774 (8,2%)
• Moderate	4.703 (21,9%)
• Mild	7.758 (36%)
• No anaemia	7.288 (33,9%)
Median Haematocrit, % (IQR)***	34,2% (30,2–38,3)
Median Mean Corpuscular Volume, fL (IQR)****	82,9 (77,2–87,7)
Median Mean Corpuscular Haemoglobin, pg/cell (IQR)†	27 (24,7–29)
Median White Blood Cells, x 10^9^/l (IQR)††	4,55 (3,65–5,80)
Leukopenia, n° (%)	7.578 (35,8%)
Median Platelet count, x 10^9^/l (IQR)†††	230 (179–296)
Thrombocytopenia, n° (%)	609 (2,9%)
Any cytopenia, n° (%)††††	8.494 (40,1%)

* n = 21.164

** n = 21.523

*** n = 21.135

**** n = 21.101

† n = 21.111

†† n = 21.176

††† n = 21.180

†††† n = 21.166

In Table 3 the frequency of different features associated with severe/moderate anaemia, leukopenia and thrombocytopenia, are shown with relative crude (COR) and adjusted (AOR) odds ratios.

**Table 3:** Factors associated with anaemia, leukopenia and thrombocytopenia (univariate and multivariate analyses).

	Anaemia severe/moderate, % (n)				Leukopenia, % (n)				Thrombocytopenia, % (n)			
	Yes	No	COR (95% CI)	AOR (95% CI)	Yes	No	COR (95% CI)	AOR (95% CI)	Yes	No	COR (95% CI)	AOR (95% CI)
**Gender**								+				
M	21,1% (1.717)	78,9% (6.417)	2,06 * (1,93–2,19)	2,92 * (2,66–3,21)	33,9% (2.709)	66,1% (5.289)	1,14 (1,07–1,21)	1,32 (1,22–1,43)	3,9% (310)	96,1% (7.690)	1,70 * (1,46–1,99)	1,39 * (1,13–1,71)
F	35,6% (4.760)	64,4% (8.629)			36,9% (4.869)	63,1% (8.309)			2,3% (299)	97,7% (12.881)		
**Nutritional status**												
Malnourished	48,3% (1.849)	51,7% (1.981)	2,91 * (2,70–3,13)	2,30 * (2,08–2,54)	34,6% (1.313)	65,4% (2.482)	0,90 (0,84–0,97)	0,78 * (0,71–0,86)	3,1% (119)	96,9% (3.676)	1,11 (0,90–1,37)	0,90 (0,69–1,17)
Not malnourished	24,3% (3.556)	75,7% (11.105)			36,8% (5.356)	63,2% (9.198)			2,8% (410)	97,2% (14.148)		
**Clinical status**												
WHO I-II	24,8% (3.085)	75,2% (9.370)	2,78 * (2,58–2,98)	2,11 * (1,92–2,32)	37,3% (4.618)	62,7% (7.748)	0,96 (0,89–1,03)	0,88 (0,80–0,96)	2,8% (350)	97,2% (12.028)	1,22 (1,00–1,48)	1,00 (0,78–1,28)
WHO III-IV	47,8% (2.084)	52,2% (2.276)			36,4% (1.575)	63,6% (2.747)			3,4% (148)	96,6% (4.170)		
**Immunological status**												
CD4+ < 200	40,2% (3.637)	59,8% (5.400)	2,28 * (2,15–2,42)	1,95 * (1,79–2,13)	44,9% (3.998)	55,1% (4.906)	1,97 * (1,86–20,9)	2,24 * (2,07–2,43)	4,0% (353)	96,0% (8.545)	1,94 * (1,64–2,28)	1,82 * (1,47–2,26)
CD4+ > 200	22,7% (2.840)	77,3% (9.646)			29,2% (3.580)	70,8% (8.692)			2,1% (256)	97,9% (12.026)		
**Virological status**												
VL < 1.000	20,7% (422)	79,3% (1.616)	1,72 * (1,54–1,93)	1,51 * (1,30–1,74)	65,7% (1.333)	34,3% (695)	1,13 (1,02–1,24)	0,99 (0,88–1,12)	2,1% (43)	97,9% (1.983)	1,46 (1,07–2,01)	0,97 (0,68–1,39)
VL > 1.000	31,1% (4.510)	68,9% (9.998)			62,9% (9.085)	37,1% (5.360)			3,1% (446)	96,9% (14.007)		

* P<0,005

Severe and moderate anaemia were observed in 1.174 (8,2%) and 4.703 (21,9%) patients respectively, involving on the whole more than one-quarter of the patients; almost one-third of the patients had Hb levels between 10 and 12 mg/dL whereas quite the same proportion of patients had normal Hb values.

Significant associations between severe/moderate anaemia and female sex, malnutrition, low CD4+ count, WHO stage III or IV and VL > 1000 copies/mL were observed using univariate analysis (Table 3). By multivariate analysis, all of these variables remained independently associated with severe/moderate anaemia. It should be noted that severe/moderate anaemia was also present in around one-quarter of patients with normal nutritional status, or with CD4+ count > 200 cells/mm^3^, or with better WHO clinical stage, and around one in five patients with VL<1.000 copies/mL.

RBC features of the patients and mean differences according to BMI, CD4+ count, and VL classes are shown in Table 4. Mean haematocrit (Hct) was 34,2% (SD: ±6,0) and was significantly lower in patients with malnutrition, immunological impairment and VL > 1.000 copies/mL. Mean MCV was 82,3 fL (SD: ± 8,6) and was significantly higher only in patients with VL >1000 copies/mL. No association was observed with nutritional and immunological status. The mean value of the Mean Corpuscular Haemoglobin (MCH) was 26,8 pg/cell (SD: ± 3,5); MCH was significantly lower in malnourished patients and patients with VL > 1000 copies/mL. No association was observed with immunological impairment. Median WBC in our cohort was 4,55 x 10^9^/l (IQR 3,65–5,80); among 21.176 patients with WBC results, more than one-third had leukopenia. In the multivariate analysis, only female sex and CD4+ count < 200 cell/ml were strong factors independently associated with leukopenia (Table 3). Median Plt count in our cohort was 230 x 10^9^/l (IQR 179–296). Among 21.180 evaluable patients, only 609 (2,9%) were thrombocytopenic. Univariate analysis showed a significant association between thrombocytopenia and male sex, immunological impairment and higher VL measurement. In multivariate analysis, only male sex and CD4+ count < 200 cell/ml were independently associated with thrombocytopenia—no association with WHO clinical stage or nutritional status was observed (Table 3).

**Table 4:** Variation of red cell indices according to BMI, CD4+ count and VL.

	All		BMI < 18,5		BMI > 18,5		P value	CD4+ count < 200		CD4+ count > 200		P value	VL < 1000		VL > 1000		P value
	Mean	SD	Mean	SD	Mean	SD		Mean	SD	Mean	SD		Mean	SD	Mean	SD	
*Hb (g/dL) n = 21*.*523*	11,1	±2,1	10,1	±2,1	11,4	±2,0	<0,0001	10,6	±2,1	11,5	±2,0	<0,0001	11,0	±2,1	11,6	±2,0	0,045
*Hematocrit (%) n = 21*.*135*	34,2	±6,0	31,5	±6,1	35,0	±5,7	<0,0001	32,7	±6,2	35,3	±5,6	<0,0001	34,0	±6,0	35,3	±5,5	<0,0001
*MCV(fL) n = 21*.*101*	82,3	±8,6	82,4	±8,7	82,2	±8,4	0,315	82,5	±8,5	82,2	±8,6	0,024	82,0	±8,3	85,4	±10,3	<0,0001
*MCH (pg/cell) n = 21*.*111*	26,8	±3,5	26,5	±3,5	26,8	±3,4	<0,0001	26,7	±3,4	26,8	±3,5	0,076	26,7	±3,3	28,2	±4,3	<0,0001

Table 5 reports the incidence of any cytopenia (anaemia and/or leukopenia and/or thrombocytopenia) and correlation with clinical, nutritional, immunological and virologic conditions. Among 21.166 fully evaluable patients, 8.494 (40,1%) had at least one cytopenia. By univariate analysis, a significant association between cytopenia and female sex, malnutrition, WHO stage III or IV, and immunological impairment was recorded. In multivariate analysis, all of these factors remained independently associated with cytopenia (Table 5). No association with VL was observed. It is however noteworthy that cytopenia was also present in 33,9% of patients with normal nutritional status, 33,9% of patients with CD4+ count > 200 cells/mm^3^, 31,7% of patients with better WHO clinical stage and 40,2% of patients with VL <1.000 copies/mL.

**Table 5:** Factors associated with any cytopenia (univariate and multivariate analyses).

	Any Cytopenia (n, %)			
	Yes	No	COR (95% CI)	AOR (95% CI)
**Gender**				
M	31,6% (2.527)	68,4% (5.468)	1,79 * (1,69–1,90)	2,48 * (2,28–2,70)
F	45,3% (5.967)	54,7% (7.204)		
**Nutritional status**				
Malnourished	60,1% (2.281)	39,9% (1.514)	2,93 * (2,72–3,16)	2,33 * (2,12–2,57)
Not malnourished	33,9% (4.931)	66,1% (9.613)		
**Clinical status**				
WHO I-II	33,9% (4.187)	66,1% (8.178)	2,81 * (2,62–3,02)	2,09 * (1,911–2,30)
WHO III-IV	59,0% (2.549)	41,0% (1.770)		
**Immunological status**				
CD4+ < 200	51,8% (4.608)	48,2% (4.289)	2,31 * (2,19–2,45)	2,15 * (1,98–2,34)
CD4+ > 200	31,7% (3.886)	68,3% (8.383)		
**Virological status**				
VL < 1.000	40,2% (814)	59,8% (1.211)	1,02 (0,92–1,12)	0,97 (0,86–1,10)
VL > 1.000	40,7% (5.875)	59,3% (8.564)		

* P<0,005

### Survival analysis

**Fig 1:**
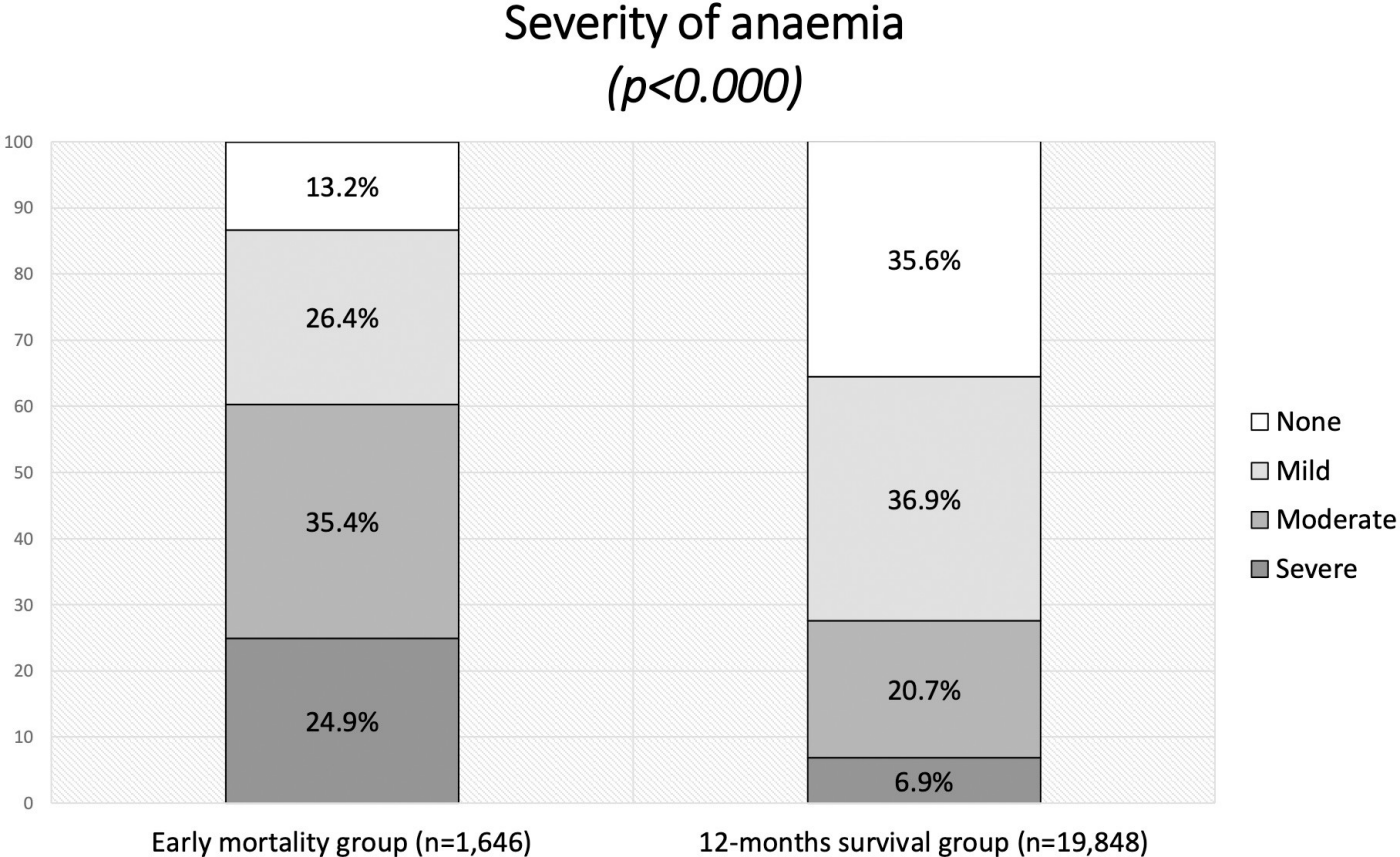
Severity of anaemia in early mortality and 12-month survival groups.

**Fig 2:**
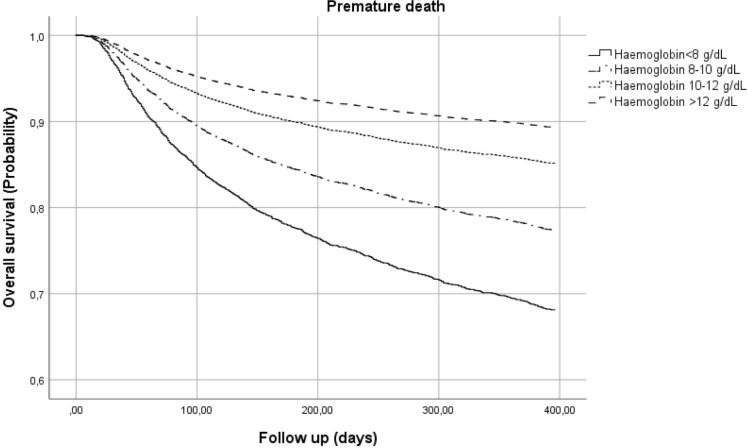
Risk of premature death by haemoglobin class.

An overall number of 1.725 subjects (7,6% of the entire sample) died within 12 months of the beginning of treatment. A comparison of initial features showed that CD4+ cell count, Hb and BMI at the baseline were significantly lower in patients with premature deaths, whereas VL was higher, as illustrated in Table 6. Among those patients who died within one year, 98,2% started ARV treatment, with an average time of therapy of 4,3 (SD ± 9,3) months. In the multivariate Cox analysis adjusted for BMI, CD4+ count and VL, the early mortality group showed higher rates of anaemia, with a significant difference in the severity of the disease, when compared with the group of patients who survived to one year from enrolment (p<0,001) (Fig 1). The risk of premature death progressively increased with the severity of anaemia: HR 1,425 [95% CI: 1,170–1,737] for mild anaemia, HR 2.271 [95%CI 1.872–2.756] for moderate anaemia and HR 3.406 [95%CI 2.762–4.200] for severe anaemia, as shown in Fig 2 and Table 7.

**Table 6:** Baseline profile of patients with premature death.

	Died in the first year of treatment		Survived in the first year of treatment		T	P value
	Mean	SD	Mean	SD		
Hb, g/dL (n)	9,6 (1.646)	±2,0	11,2 (19.848)	±2,1	-30,693	<0,0001
BMI, kg/m^2^ (n)	19,3 (1.441)	±3,4	21,8 (17.539)	±4,0	-25,549	<0,0001
CD4+ cell count, cells/mm^3^ (n)	140	±141*	263	±18	-28,577	<0,0001
Viral Load, log10 copies/ml (n)	4,6 (1.314)	±1,2	4,1 (15.662)	±1,3	13,131	<0,0001

* The reason for such an abnormal SD could be the high variability of the cohort, so that we had extremely higher values of CD4+ that impacted on the variance and hence the SD.

**Table 7:** Multivariate model for overall survival at 1 year.

	B	SE	Wald	gl	HR^§^	HR 95%CI		
						Lower	Upper	P value
Hb <8 g/dL versus Hb >12 g/dL	1.226	.107	131.295	1	3.406	2.762	4.200	<0.0001
Hb 8–10 g/dL versus Hb >12 g/dL	.820	.099	69.251	1	2.271	1.872	2.756	<0.0001
Hb 10–12 g/dL versus Hb >12 g/dL	.355	.101	12.394	1	1.425	1.170	1.737	<0.0001

§ multivariate analysis adjusted for CD4+ count, Viral Load and Body Mass Index

**Fig 3:**
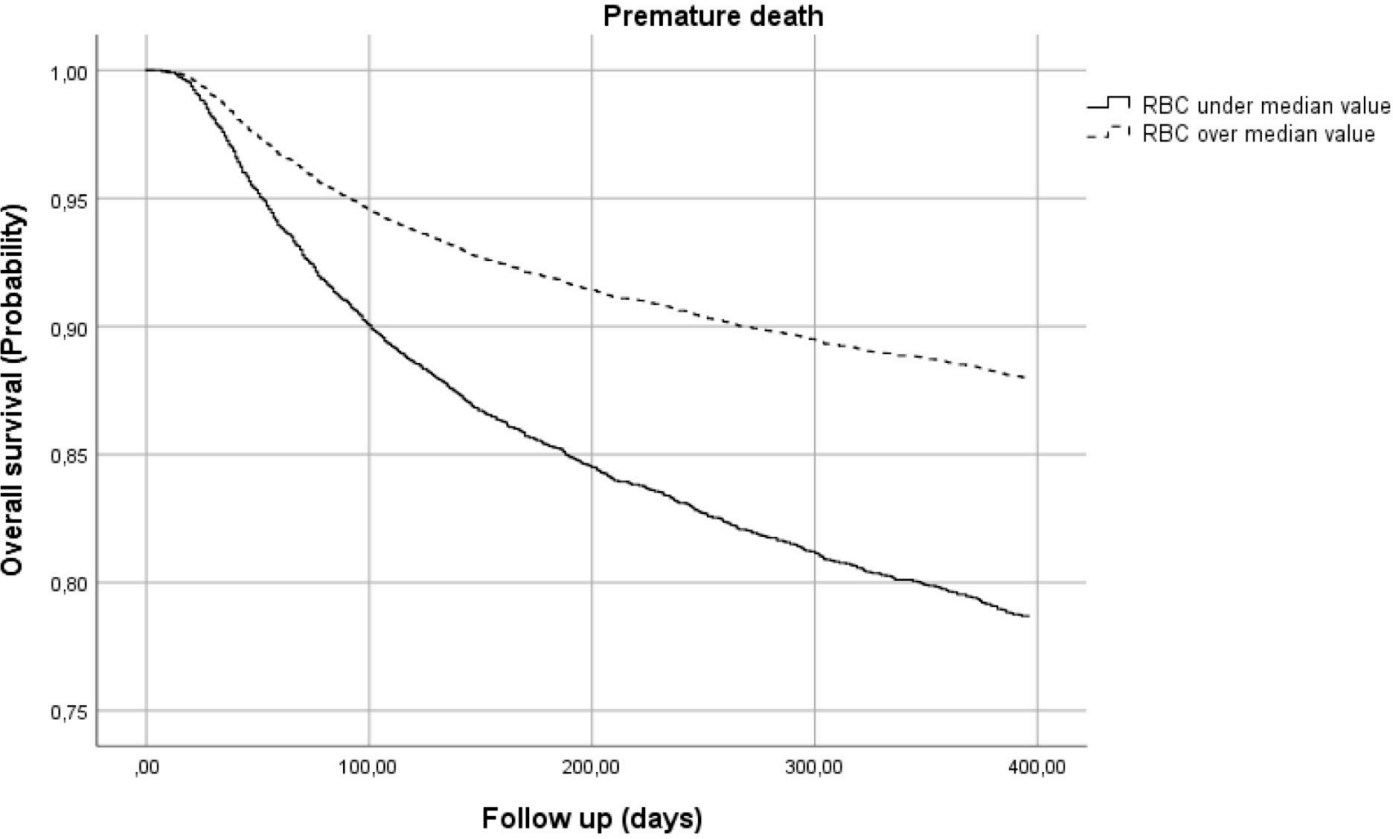
Risk of premature death by red blood cell (RBC) count.

**Fig 4:**
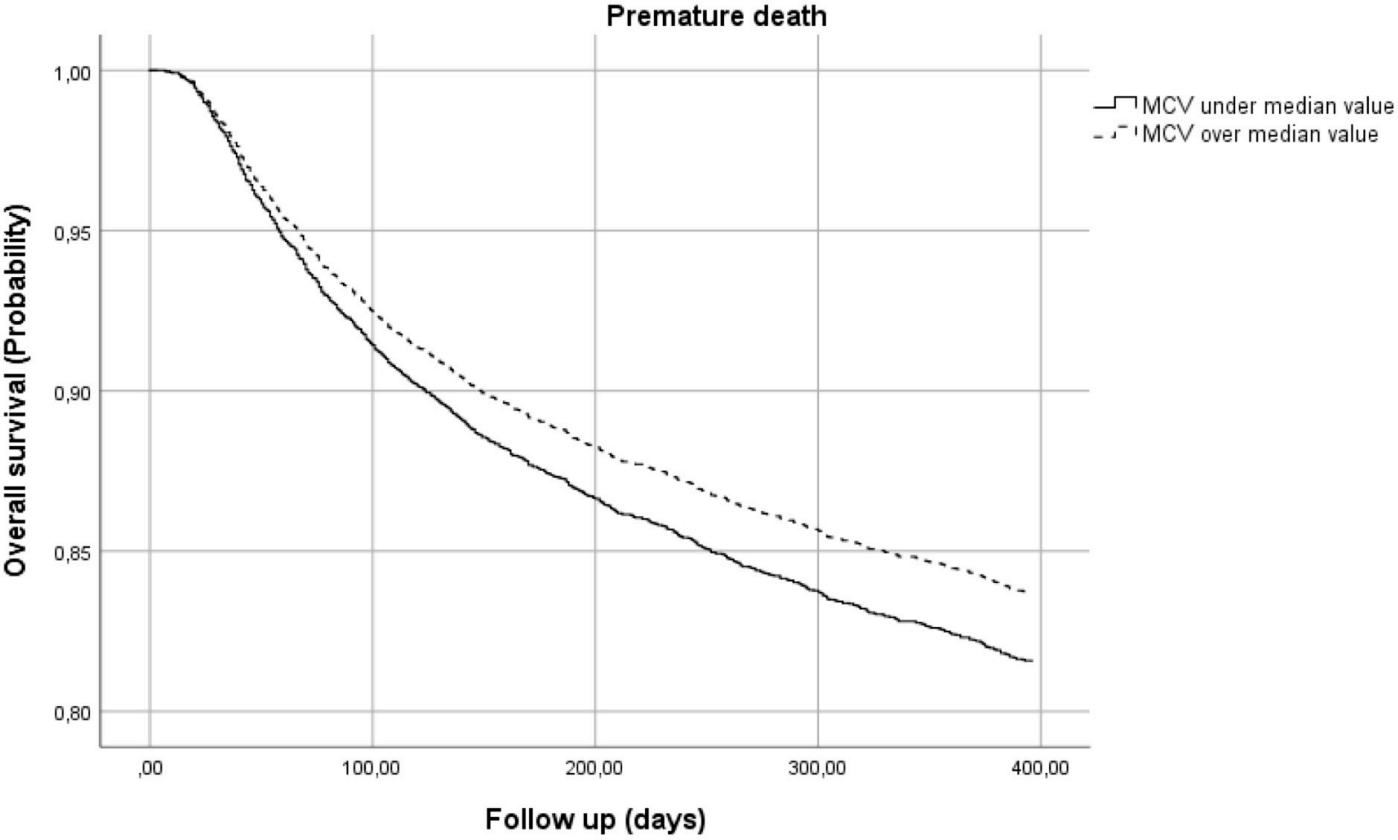
Risk of premature death by mean corpuscular value (MCV).

In addition, data for patients with an RBC count over or under the median value of 4,2 million/mm^3^ were analysed. The risk of premature death for patients with lower RBC increased by nearly 10% compared to patients with higher baseline RBC (Fig 3). Also, MCV values influenced early mortality (Fig 4): patients with lower MCV at enrolment showed a slightly increased risk of premature death (HR 1.146; 95%CI 1.017–1.291).

**Fig 5:**
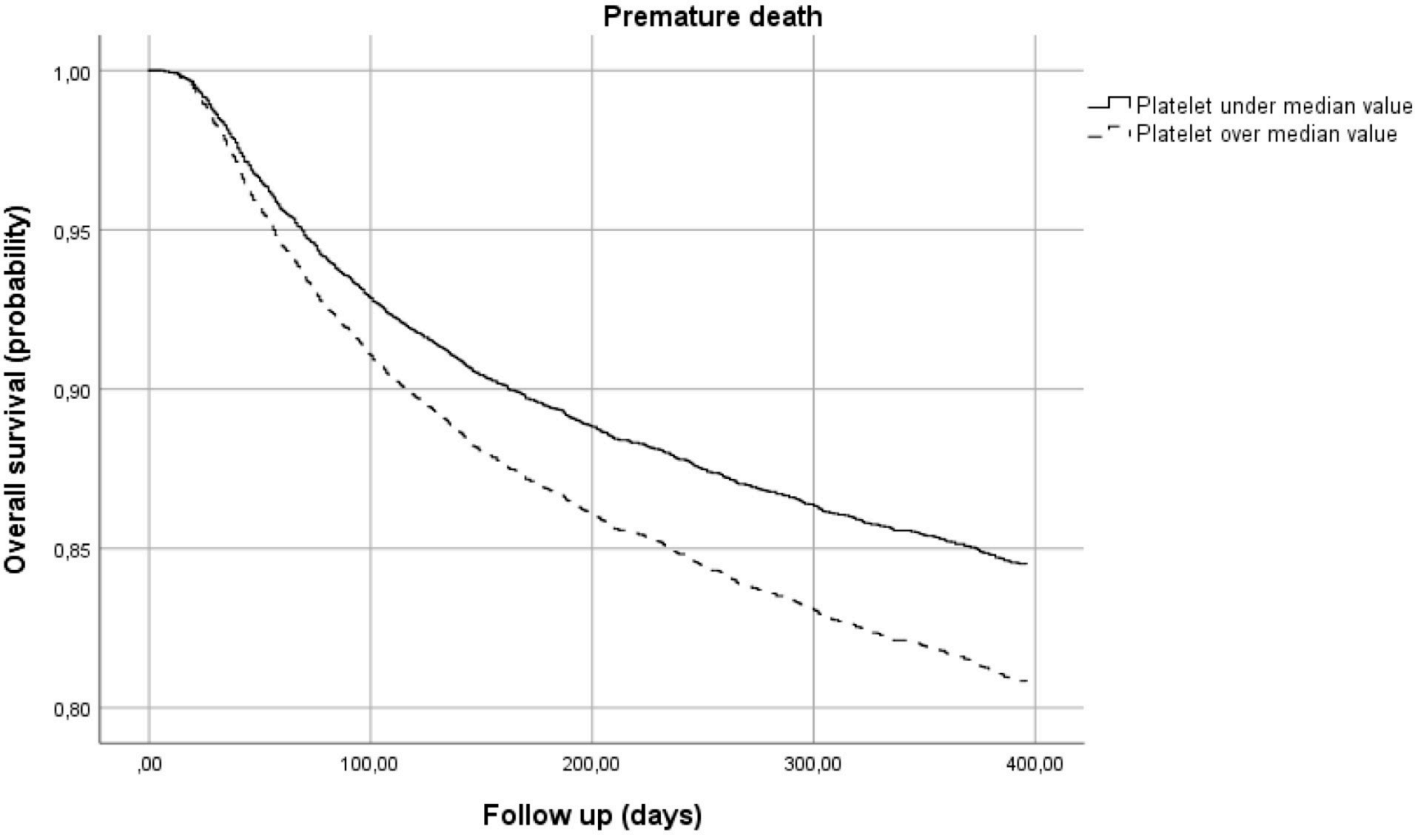
Risk of premature death by platelet count.

Fig 5 shows the survival curves of patients with different Plt counts, independent of CD4+ count, BMI and VL. The probability of survival at one year for patients with a Plt count higher than the median value (230 x 10^9^/l) was slightly but significantly lower than for those with a Plt count under the median value.

## Discussion

Anaemia is known to be associated with HIV infection at diagnosis [20]. However, full peripheral blood evaluation is not routinely performed in many African countries, where HIV infection has an unusually high prevalence [1]. As a consequence, data regarding the true incidence of anaemia and other haematologic alterations in the real-life setting of PLWH in resource-limited countries is lacking. To our best knowledge, this is the first attempt to evaluate haematologic alterations at baseline of ART in a vast, unselected real-life cohort of naive PLWH in Africa.

Our analysis shows that haematological alterations are common in HIV patients and are correlated with early mortality. In particular, it is worth to notice the relatively high prevalence of abnormalities in patients in better clinical status that could represent a group of patients valuable for more strict and careful monitoring.

### Baseline haematological features and cytopenia

In our cohort, anaemia was a common condition, with a higher incidence in women and patients with advanced HIV disease as expected. In particular, it is worth to notice that 86,8% of patients who died during the first year of treatment were anaemic.

Leukopenia was also common, but this may be partially due to the lymphocytopenia associated with HIV infection. It should be noted that although a low prevalence of thrombocytopenia was reported in our cohort (2,9%), this could be a significant concern in high HIV-burden countries due to the considerable number of HIV cases.

Rates of anaemia, leukopenia and thrombocytopenia were lower than those described in other countries [21]. However, they are in line with studies performed in different settings [20, 22]. The discordance with Firnhaber et al. [21] could be explained through differences in the size and selection of the sample. Also, Firnhaber and colleagues evaluated patients from several countries with different resource availabilities [21]; the strong association of haematologic features with general, clinical and social characteristic of the population could justify such differences and support our results.

As expected, RBC features were variously associated with general conditions and HIV-infection progress.

As other authors already observed [23], a comprehensive haematologic analyses showed the high prevalence of one or more cytopenia of any type (40,1%) in patients with severe HIV disease. In our opinion, however, this is only one aspect of the problem. Our study further noted a high frequency of haematologic alterations in patients otherwise in generally good condition. Among patients without malnutrition, in a better WHO clinical stage, CD4+ > 200 and lower VL, 22–24% were anaemic, and 31–40% had some cytopenia.

### Survival analysis

The association of cytopenia, especially anaemia, with poor outcomes in PLWH is well known [24–27]. Our early survival analysis emphasizes the significant prognostic value of different hematologic alterations at baseline.

Some studies have assessed the causes of death in HIV+ patients [28, 29]. We are aware that other, often fatal, comorbidities may influence haematological features. For example, anaemia is one of the leading clinical signs of TB coinfection and its specific inflammatory profile [13, 30, 31]. Therefore, we hypothesize that some early deaths could be related to an HIV/TB coinfection which induces a concomitant severe anaemic status. Also, viral infections are among the leading causes of fatal events in HIV patients [29] and, at the same time are known to interact with bone marrow functions to cause different degrees of haematopoietic suppression [32]. In particular, the interaction of CMV with haematopoietic functions and the resulting impact on mortality could be significant [33].

Finally, cancers are reported among the leading causes of death in HIV+ patients, and these may also be related to haematological alterations [34, 35].

The above considerations could explain our results related to haematological alterations and mortality in HIV patients—in particular, the correlations with Hb, RBC, MVC, and Plt count.

The increased early mortality rate in patients with a higher neutrophil count is a counterintuitive finding but may be related to undiagnosed bacterial infections at the time of ART initiation. Moreover, a higher mortality rate was observed in HIV+ patients with concomitant cryptococcal meningitis and neutrophilia [36]. Cryptococcal meningitis could also contribute to explain our findings.

### Limitations of the study

We are aware of certain limitations in our study. Although the use of data from a vast real-life setting is one a strength, it is limited by the incompleteness of data collected retrospectively from routine medical records. A further challenge was the absence of some clinical and laboratory information that would have provided a more informative picture of haematologic conditions in our patients. In particular, the possible presence of comorbidities, the study of serum iron, folate and cyanocobalamin, would have given more extensive knowledge.

### Public health and clinical impact

Our results encourage a more accurate evaluation of the haematologic pattern in PLWH. In particular, our analysis supports the need for haematologic screening with full blood count before starting ART. Some authors have already underlined the feasibility of haemoglobin measurement as a low-cost and feasible proxy for severity of clinical status in HIV+ patients [37, 38]. Moreover, the International Council for Standardization in Hematology argues for more comprehensive haematological assessment at all levels of care [39].

Our findings provide additional evidence in support of a more accurate clinical focus on haematologic alteration in HIV+ patients, even when patients present with apparently good health status. Furthermore, results from the DREAM program showed the feasibility and the effectiveness of a complete haematologic assessment in a resource-limited setting.

## Conclusion

In conclusion, from our large cohort of patients in two resource-limited countries, hematologic alterations at the baseline of ART were common and associated with other clinical and biologic findings of prognostic value. A long-term survival analysis of PLWH according to different haematologic results could provide further valuable information in this sense, especially in the absence of other risk factors. However, to overcome the limits of present retrospective analysis and confirm the results, a prospective study is warranted.
